# Interdisciplinary German clinical practice guidelines on the management of type B aortic dissection

**DOI:** 10.1007/s00772-023-00995-5

**Published:** 2023-04-24

**Authors:** A. Oberhuber, A. Raddatz, S. Betge, C. Ploenes, W. Ito, R. A. Janosi, C. Ott, E. Langheim, M. Czerny, R. Puls, A. Maßmann, K. Zeyer, H. Schelzig

**Affiliations:** 1 German Society of Vascular Surgery and Vascular Medicine (DGG); Department of Vascular and Endovascular Surgery, University Hospital of Münster, Münster, Germany; 2 German Society of Anaesthesiology and Intensive Care Medicine (DGAI); Department of Anaesthesiology, Critical Care and Pain Medicine, Saarland University Hospital, Homburg, Germany; 3 German Society of Angiology and Vascular Medicine (DGG); Department of Internal Medicine and Angiology, Helios Hospital Salzgitter, Salzgitter, Germany; 4 German Society of Geriatrics (DGG); Department of Angiology, Schön Klinik Düsseldorf, Düsseldorf, Germany; 5 German Society of Internal Medicine (GSIM) (DGIM); cardiovascular center Oberallgäu Kempten, Hospital Kempten, Kempten, Germany; 6 German Cardiac Society (DGK); Department of Cardiology and Angiology, University Hospital Essen, Essen, Germany; 7 German Society of Nephrology (DGfN); Department of Nephrology and Hypertension, Friedrich-Alexander University Erlangen-Nürnberg, Erlangen, Germany; 8 German Society of prevention and rehabilitation of cardiovascular diseaese (DGPR), Reha Center Seehof, Teltow, Germany; 9 German Society of Thoracic and Cardiovascular Surgery (DGTHG), Department University Heart Center Freiburg – Bad Krozingen, Freiburg, Germany; 10 German Radiologic Society (DRG); Institute of Diagnostic an Interventional Radiology and Neuroradiology, Helios Klinikum Erfurt, Erfurt, Germany; 11 German Society of Interventional Radiology (DeGIR); Department of Diagnostic an Interventional Radiology, Saarland University Hospital, Homburg, Germany; 12 Department of Nephrology and Hypertension, Paracelsus Medical University, Nürnberg, Germany; 13 Albert Ludwigs University Freiburg, Freiburg, Germany; 14 Marfanhilfe e. V., Weiden, Germany; 15 German Society of Surgery (DGCH); Department of Vascular and Endovascular Surgery, University Hospital of Düsseldorf, Düsseldorf, Germany

## Introduction

Aortic dissection is a rare but life-threatening disease, in which an intimal tear causes bleeding inside the aortic wall. Depending on the localization of the entry tear, aortic dissections were divided into type A (involvement of the ascending aorta, independent of the entry tear but normally in the ascending aorta) and type B dissections (entry tear distal to the left subclavian artery). Type A aortic dissections should be managed as fast as possible with an open repair; the corresponding guidelines for performing open repair are published elsewhere. The uncomplicated type B aortic dissection is normally managed by conservative treatment. The goal of these renewed guidelines is to outline the pathophysiology, epidemiology and management of type B aortic dissections, including imaging, treatment, aftercare and rehabilitation.

## Methods

The first version of the guidelines was published in 2018 and has now become outdated; an update of the guidelines became necessary. This version is not only an actualized version of the old guidelines but also extends the document by providing new sections.

The guidelines were developed under the supervision and guidelines of the Association of the Scientific Medical Societies in Germany (AWMF). The AWMF distinguishes between several classes of guidelines (https://www.awmf.org/en/clinical-practice-guidelines/awmf-guidance/awmf-guidelines-register.html). The guidelines were developed as class S2k guidelines, which are consensus-based guidelines. For this type of guidelines, all relevant scientific societies should be involved and all recommendations must be consented to by participants of conferences or the Delphi technique. In addition, patient representatives were involved in these guidelines. Due to the coronavirus disease 2019 (COVID-19) pandemic all conferences were held online. The original version of these guidelines was published online in German in May 2022 on the homepage of the AWMF [1].

### Consensus process

In an initial meeting, the topics and aims of the guidelines were discussed and defined. Thereafter, different working groups were defined and topics were assigned.

Based on the nature of class S2k guidelines, no structured literature search was used; however, all relevant papers from 2017–2022 were searched across different databases (Medline, Cochrane Database and EMBASE), including international guidelines. Based on this literature review, a first draft was written. This draft was review by all members and discussed until a consensus was found. The recommendations were then defined, and an online Delphi procedure was used for the consensus process. Consent between the 13 involved societies > 75% was defined as strong consent and > 95% as very strong consent. The individual strength of consent in each recommendation is presented with the relevant recommendations below. The entire document was reviewed and consented to by the executive board of each scientific society involved.

**Fig. 1:**
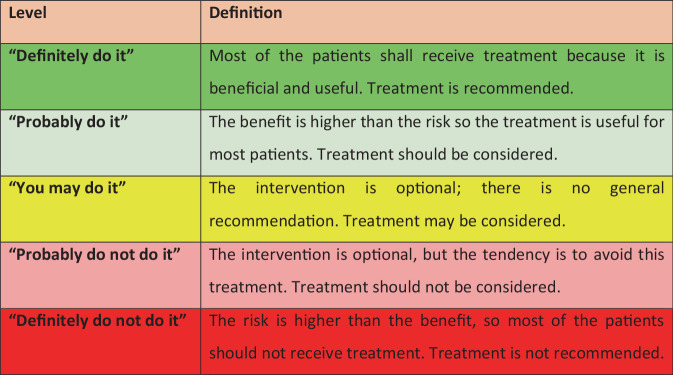
Table showing the levels of the recommendations

The recommendations are divided into five groups (Fig. 1).

The entire process was accompanied by the AWMF including all consensus conferences. Each member of the steering group was required to declare their interests, which are available online (https://www.awmf.org/fileadmin/user_upload/Leitlinien/004_D_Ges_fuer_Gefaesschirurgie/004-034i_S2k_Typ_B_Aortendissektion_2022-05.pdf).

## Definition and classification

Aortic dissection is caused by an intimal tear that leads to bleeding inside the aortic wall. At this primary entry site, the aortic lumen is divided into a new false lumen and the true lumen. Intramural hematoma (IMH) is a type of dissection in which bleeding in the aortic wall occurs due to a rupture of the vasa vasorum or dissection without re-entry and complete thrombosis of the false lumen [2]. An IMH can pass into an aortic dissection, and both entities can occur at the same time; therefore, patients do not typically present with two diseases and instead present with one disease with two different appearances [3].

### Classification according to localization of the primary entry

The widest accepted classifications for dissections were published over 50 years ago by DeBakey et al. (1965) [4] and Dailey et al. (1970) [5]. The DeBakey classification differentiates between dissections according to extension, the Stanford classification distinguishes between dissections whether the ascending aorta or descending aorta is involved, regardless of the localization of the entry (Table 1).

**Table 1:** DeBakey and Stanford classifications for aortic dissections

DeBakey classification	Stanford classification
Type I: primary entry in the ascending aorta with involvement of the aortic arch and the descending aorta	Stanford A: all dissections with involvement of the ascending aorta regardless of the localization of the primary entry. In most cases, this entry is also located in the ascending aorta
Type II: primary entry in the ascending aorta without involvement of other parts of the aorta	
Type III: primary entry in the descending aorta	Stanford B: all dissections with involvement of the descending aorta
Type IIIa: extension to the diaphragm	–
Type IIIb: extension below the diaphragm	–

In the daily routine, the Stanford classification is the mostly accepted and used classification. All dissections with involvement of the ascending aorta must be repaired, whereas type B dissections can be managed conservatively. These practical guidelines only address type B dissections.

This classification does not represent all patients, therefore in the last years the term non-A-non‑B dissection was introduced. This represents all patients with involvement of the aortic arch, regardless of whether the entry is in the descending aorta or in the aortic arch.

### Classification according to time of onset

Another method of classifying type B aortic dissections is to use the time of onset of symptoms. The authors propose, in addition to other similar classifications, [6, 7] the following classification ([8]; Table 2).

**Table 2:** Chronologic classification of type B aortic dissections

Classification	Time from symptom onset
Acute	1–14 days
Subacute	15–90 days
Chronic	> 90 days

### Classification according to symptoms

Classification according to symptoms or time of onset of symptoms is not helpful for treatment decision-making. In a 2013 expert consensus document, [7] type B aortic dissections were divided into asymptomatic and symptomatic dissections:

- Malperfusion of the aortic branches (spinal, iliac, visceral, renal).
- Refractory hypertension: hypertension despite three different classes of antihypertensive medication at the maximum dosage. It is a sign of instability or renal malperfusion.
- Augmentation of the periaortic hematoma and pleural effusion in two consecutive CT scans are signs of an impending rupture.
- Patients with false lumen rupture, circulatory instability and severe hypotension should be considered as being in severe life-threatening conditions.

### Complex classifications

All classifications lack consideration of the complexity of the disease and present no direct recommendations for treatment.

Dake et al. [9] defined the following classification, which they refer to as DISSECT, that considers both morphological and clinical criteria:

- **D**uration
- **I**ntimal tear
- **S**ize
- **S**egmental **e**xtent
- **C**linical complication
- **T**hrombosis of the false lumen

More recently, the TEM classification [10], similarly to the oncological TNM classification, considers the three following criteria:

- Type (T): A, B and non-A-non‑B aortic dissections
- Entry (E): localized in Ishimaru zones 0–3 [11]
- Malperfusion (M):
  - M0 – no malperfusion
  - M1 – coronary malperfusion
  - M2 – supra-aortic malperfusion
  - M3 – spinal, visceral or iliac malperfusion
  - (−) no symptoms
  - (+) symptoms

The disadvantage of the TEM classification is that the distal extension is not included.

The Society for Vascular Surgery (SVS) and the Society of Thoracic Surgeons (STS) published new reporting standards in 2020 [8]. In these standards, extension is still based on the Ishimaru classification but the distal extension is also included. The disadvantage of these standards is that the classifications only distinguish between type A and type B aortic dissections. Type B dissections are defined as all dissections where the primary entry is not in zone 0.

All new classifications must be evaluated based on their use in daily routine; otherwise, no statements can be made on their clinical benefits.

#### Statement

- The Stanford classification is the most used classification with the **highest** clinical benefit.
- The TEM classification has advantages for treatment decision**-making;** the reporting standards have advantages during the follow-up because of the inclusion of the distal extension.
- Other classifications are used regarding clinical aspects and time between onset of symptoms and presentation.

## Epidemiology and pathophysiology

### Incidence

Olsson et al. [12] found, in their 2003 population-based study, an incidence of thoracic aortic disease of 16.3 per 100,000 male inhabitants and 9.1 per 100,000 per female inhabitants in Sweden; 40% of these cases were aortic dissections. In the OXVASC study, the incidence was approximately 6/100,000 between 2002 and 2012. Type B dissections were 28.8% less common than type A aortic dissections (71.2%) [13].

Additionally, in the Malmö Diet and Cancer Study (MDCS), type A dissections (58%) were more frequent than type B dissections [14]. The incidence was blood pressure-dependent. In patients with arterial hypertension, the incidence was 21/100,000, whereas in patients without arterial hypertension the incidence was only 5/100,000. In patients between 65 and 75 years old, the incidence was 35/100,000 [13].

In a large Swedish population-based study (2002–2016), the annual incidence was 7.2/100,000, and men were affected more often than women (9.1 vs. 5.4) [15]; however, women from the sample were shown to experience higher mortality rates [13, 16–18].

A major barrier to understanding true incidence rates is the estimated number of unknown cases. An autopsy study revealed that 50% of type A dissections in emergency departments remain undetected [19]. In another autopsy study, which was carried out in Hungary where all sudden deaths undergo an autopsy, it was shown that only 6% of the patients with ruptured aortic dissection reached hospital [20].

A higher or altered incidence during the COVID 19 pandemic or in association with the severe acute respiratory coronavirus 2 is not reported.

### Risk factors and pathophysiology

In the OXVASC trial, 67.3% of the patients had arterial hypertension and 61.5% were smokers [13]. In the MDCS trial, smoking and arterial hypertension were also shown to be significant risk factors for aortic dissection; however, additional risk factors included increasing age, male sex and low apolipoprotein A levels [14].

In the International Registry of Aortic Dissection (IRAD) trial, arterial hypertension was associated with aortic dissection in 76.6% of cases. Other risk factors were atherosclerosis, aortic aneurysm and previous cardiac operations [17].

Studies have presented a clear genetic predisposition for aortic dissection. In the IRAD trial, 5% (53 out of 1049) of patients had a diagnosis of Marfan syndrome. These patients were significantly younger and displayed significantly fewer cases of arterial hypertension and atherosclerosis. The predominant dissections in Marfan syndrome patients were type A dissections (76% vs. 62% in non-Marfan patients) [21].

In addition to Marfan syndrome, other syndromic diseases are Loeys-Dietz syndrome and Ehlers-Danlos syndrome [22]. In contrast, other genetic variations, which are referred to as non-syndromic diseases, can be predictors of familial aortic dissections. Several of these gene mutations are known, which include the following: *ACTA2, MYH11, MYLK, PRKG1, MAT2A, MFAP5, FOXE3, THSD4, SMAD *and* LOX* [2, 23]. All of these genetic conditions code for components of the aortic wall (e.g. fibrillin‑1 in Marfan patients) or wall-modifying proteins (e.g. LOX) [24].

A first multigene screening test, including 21 candidate genes, was published in 2015. No other results using this test have yet been published [25].

Patients with aortic dissections present with altered microstructures of their extracellular matrix, which results in the degradation of elastic fibers [26–28]. The dissected wall presents increased inflammation and the infiltration of immune cells [29]. The interaction between the extracellular matrix proteins and growth factors such as TGF-beta or BMP plays an important role in developing dissection and aneurysms. This interaction results in an extensive release of TGF-beta, caused either by incorrect sequestration of fibrillin‑1 in Marfan patients or mutation of the TGF-beta signal cascade in Loeys-Dietz patients [30–33]. Although previous research has provided insights into fundamental pathophysiological processes, clear triggers of aortic dissection have not yet been found [2, 34–36].

Ma et al. [37] investigated 32 relatives of 100 patients with aortic dissection. They found a 2.8-fold increase in the patients’ relatives’ annual risk of de novo aortic dissection. A positive family history was the most relevant risk factor for aortic dissections.

In young patients, other risk factors, such as substance abuse, must be considered. Cocaine elevates the risk for type A dissections. Other triggering substances include amphetamines.

#### Statement

Risk factors for type B aortic dissections are recognized to include positive family history, male sex, smoking and arterial hypertension. In young patients, in addition to genetic background, substance abuse should be considered.

## Presenting symptoms and complications

### Presenting symptoms

Similar to acute coronary syndrome, abrupt, severe, sharp chest pain located in the anterior chest or interscapular region can be presenting symptoms for acute aortic syndrome. This term includes classical aortic dissection, intramural hematoma (IMH) and penetrating aortic ulcers (PAU).

In an analysis of the IRAD register, the following symptoms and results were noted: ([38]; Table 3).

**Table 3:** Symptoms and diagnostic findings of type B aortic dissection in the IRAD register

Symptoms	Patients with type B aortic dissection (%)
Most severe pain ever experienced	88.7
Pain in the anterior chest or interscapular region	88.7
Sudden onset of pain	85.4
Moving pain	16.8
Syncope	2–6
Arterial hypertension	64.6
Pulse deficit	26.3
Widened mediastinum	42.6

An inconspicuous chest X‑ray was found in 30% and an inconspicuous ECG was found in 40% of the cases, suggesting neither test is appropriate for aortic dissection diagnosis.

Women presented noteworthy atypical symptoms and reported less severe pain [39].

### Complications

For treatment to succeed after primary diagnosis, it is very important to distinguish between complicated and uncomplicated dissections. An international expert consensus document published a definition for a complicated type B aortic dissection that included malperfusion, refractory hypertension and impending rupture [7].

#### Malperfusion

The incidence of malperfusion of aortic side branches varies between 25% and 50%. The perfusion of the false lumen compresses the perfusion of the true lumen. In a dynamic compression, during the systole the prolapsing dissection membrane covers the ostium of the side branch, whereas in a static condition the membrane directly causes a stenosis. This narrowing can result in a thrombosis of the side branch and ischemia of the organ [40, 41]. Severe malperfusion is one of the main causes of the high morbidity and mortality of acute aortic dissection patients. In another analysis of the IRAD register (1996–2013), the incidence of complications of 1034 patients with type B aortic dissections were evaluated [41].

- acute kidney injury: 17.9%
- hypotension: 9.7%
- limb ischemia: 9.5%
- mesenteric ischemia: 7.4%
- spinal ischemia: 2.5%

These complications were independent predictors of in-hospital mortality. The odds ratio for mesenteric ischemia was 9.03 (95% CI 3.49–23.38), hypotension 6.43 (95% CI 2.18–18.98), acute kidney injury 3.61 (95% CI 1.68–7.75) and limb ischemia 3.02 (95% CI 1.05–8.68).

Although only 17% of the patients had an aortic diameter ≥ 5.5 cm at presentation, the odds ratio of 6.04 for this condition (95% CI 2.87–12.73) was remarkable high [41].

**Fig. 2:**
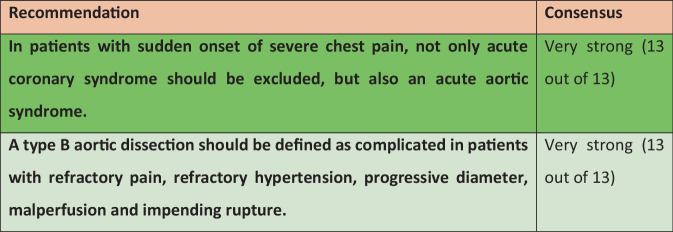
Recommendation 1: clinical presentation and complication

The criteria for a complicated acute aortic dissection type B include the provision of certain clinical and diagnostic characteristics, which include the following: ([40, 42]; Fig. 2)

- rupture, hypotension or shock
- malperfusion with:
  - visceral ischemia
  - limb ischemia
  - spinal ischemia
- high risk patients with:
  - refractory pain
  - refractory arterial hypertension
  - rapid expansion of the aortic diameter

## Diagnostic testing

The most important examination for diagnosis and treatment planning is the ECG-gated computed tomography with contrast medium. In all hemodynamically unstable patients, this tomography should occur immediately (class I, level C). If possible, a transthoracic echocardiography should also be performed immediately to assess for cardiac pump function, the aortic root and a possible involvement of the pericardial space.

**Fig. 3:**
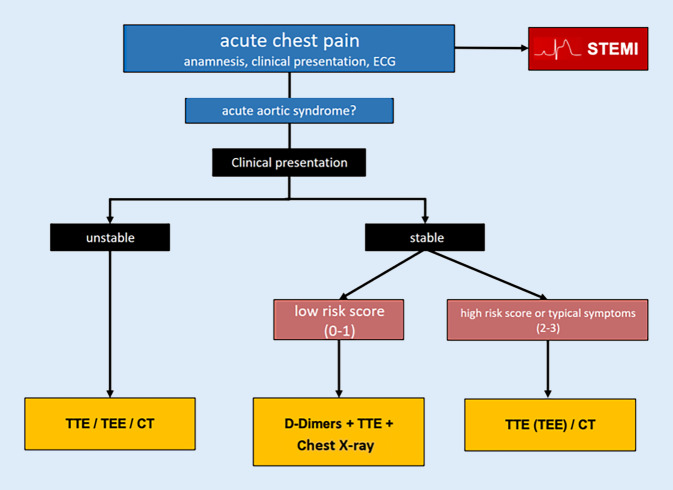
Diagnostic algorithm (modified ESC guidelines 2014) [43]. *ECG* electrocardiogram, *STEMI* ST-elevation myocardial infarction, *TTE* transthoracal echocardiography, *TEE* transesophageal echocardiography, *CT* computed tomography

For all hemodynamically stable patients with chest pain, it is important that according to a diagnostic algorithm 1) an acute aortic dissection is considered, 2) a prompt diagnostic is performed and 3) irrelevant and time-consuming diagnostics are avoided. Figure 3 presents a proposed algorithm for this process.

### Medical history

Most patients with aortic dissections have arterial hypertension; therefore, it is important to check patients’ previous medications and decompensations. Standard questions about risk factors and comorbidities are also part of the medical history. In addition to standard risk factors, genetic risk factors also play an important role, especially in younger patients. These patients should be actively asked for their known connective tissue diseases, familial clustering of dissections or other aortic pathologies.

### Presenting symptoms

The guiding symptom for aortic dissections is pain. The most depicted symptom for aortic dissections is pain in the back or in the interscapular region with abrupt onset and of sharp character. The radiation of the pain is dependent on the extension and involvement of side branches. The pain localization can migrate or radiate during the first hours after onset of symptoms.

Depending on the side branches involved and perfusion of the false lumen the following symptoms can occur: thromboembolic stroke with weakness of arms or legs, ischemic complications due to malperfusion of visceral organs and paraplegia or hemodynamic instability in cases that involve the ascending aorta, coronary arteries or rupture.

Physical examination findings can include heart murmurs, murmurs over involved side branches, pulse deficits and side differences in blood pressure. Although these guidelines deal with type B aortic dissections, a type A dissection is a classical differential diagnosis and should always be kept in mind. In patients with type A dissection, the neurological symptoms are more common and can obscure the clinical presentation [43, 44], these patients less commonly report the typical aortic pain associated with dissections (class I/level C) [44].

### Biomarkers

There is no single biomarker that can detect aortic dissection with high sensitivity and specificity. This inability makes diagnosis of aortic dissections more difficult [45].

In specialized laboratories biomarkers of aortic wall degeneration can be measured but these tests are not widely available. The measurement of the D‑dimer concentration is routine and also available as a point-of-care test. D‑dimer tests’ main limitation is their low specificity. Marill [45] found a high sensitivity of about 94%, the specificity was between 40% and 100% for the D‑dimer test. Cui et al. [46] found a sensitivity of 94.5% and specificity of 69.1%, whereas Watanabe et al. [47] found a sensitivity of 95.2% and specificity of 60.4%. A D-dimer concentration < 500 ng/ml very rarely indicates an acute aortic dissection.

These results were confirmed in a meta-analysis of 790 patients with acute chest pain. The D‑dimer test was performed within 24 h of pain onset. The sensitivity was 94% and specificity 56.8%. In this meta-analysis, an acute aortic dissection could be excluded with the D‑dimer test, but because of relatively low specificity the diagnosis could not be verified. The D‑dimer levels were not significantly different from those with pulmonary embolism.

The use of the sole measurement of the D‑dimer concentration for excluding aortic dissections leads to a high rate of unjustified CT examinations with corresponding radiation and contrast exposure.

Using a low cut-off of < 0.1 µg/ml, which was derived from a systematic review including 16 trials with 437 patients, the sensitivity to exclude an acute aortic dissection was 100% in a prospective cohort of 65 patients [48]. This approach has not yet been tested in a larger cohort.

### Scoring systems and combinations of different testing methods

**Fig. 4:**
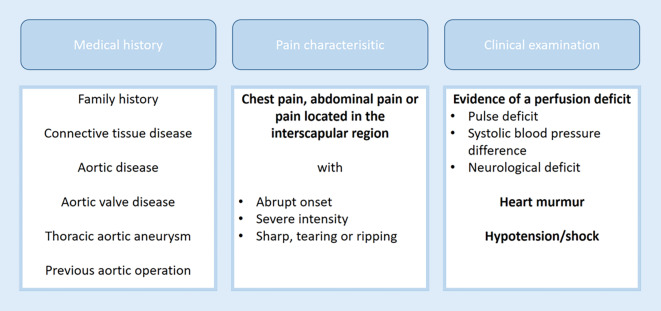
Clinical assessment of risk factors (AHA Aortic Dissection Risk Score, ADD-RS)

The 2010 ACC/AHA guidelines for thoracic aortic disease elaborated on previous information listing comorbidities, acute symptoms and examinations that correlate with a high risk of an acute aortic dissection [6]. From this list, an aortic dissection detection risk score (ADD-RS) was proposed (Fig. 4). Each group was valued with one point before risk was stratified as low (0 points), intermediate (1 point) or high (2–3 points).

In a retrospective analysis of the IRAD register including 2538 patients, only 108 patients (4.3%) had a low risk of aortic dissection [44]. In other analyses, only 5.9% with zero points in the ADD-RS had an aortic dissection [48]. An additional conventional chest X‑ray presents no significant benefit [49].

In a retrospective analysis of secondary care hospitals, hospitals that normally have a low incidence of aortic dissections, the sensitivity for acute aortic syndromes with ADD-RS ≥ 1 was 100%, but specificity was only 12.3% [50].

In the current ESC guidelines, the two first risk classes were subsumed into a low-risk class (0–1 point), and other items were added. In a prospective multicenter observational trial (ADViSED), 1850 patients with suspected acute aortic syndrome were analyzed for their AAD-RS (0–3) risk scores. Additionally, the D‑dimer concentration (cut-off value < 500 ng/ml) was analyzed. A positive D‑dimer test had a sensitivity of 96.7% and specificity of 64%. The error rate of a combination of AAD-RS = 0 and “negative” D‑dimer testing was 0.3%; the combination of AAD-RS ≤ 1 and D‑dimer testing was < 1%. The efficacy of the AAD-RS = 0 and D‑dimer testing was 15.9%; the efficacy of AAD-RS ≤ 1 and “negative” D‑dimer testing was 49.9% [51].

In a single-center retrospective analysis of 376 patients with chest pain and available D‑dimer tests, the negative predictive value for AAD-RS ≤ 1 and “negative” D‑dimer testing was 89.9% with an error rate of 1.1% [52].

A recent meta-analysis, including 9 studies and 26,958 patients with AAD-RS and 3241 patients with D‑dimer testing, found a high sensitivity for the AAD-RS alone or in combination with the D‑dimer testing [53]. In a multivariate regression analysis, six items from the ADD-RS (previous thoracic aortic aneurysm, severe intensity, abrupt onset, pulse deficit, neurological deficit and hypotension/shock) were evaluated as independent risk factors for an acute aortic syndrome. In combination with age-adjusted D‑dimer testing, these items were developed to create an easier diagnostic tool.

In a retrospective analysis of two centers with high prevalence and a prospective analysis of two centers with low prevalence, the score was externally validated. With these data, a weighting of the single items was performed: hypotension/shock equalled 2 points and all other items equalled 1 point. The new, simplified score was called AORTAs. In this score, a value ≤ 1 describes a probability of 4.6% for an acute aortic syndrome. A score ≥ 2 describes a high probability of acute aortic syndrome and justifies further evaluation with imaging [54]. This simplified score must be evaluated further in a larger prospective cohort.

Currently, no single test can identify patients with a low risk for acute aortic syndromes; additionally, combinations of different tests have not yet been evaluated enough to confidently ascertain low risk. It is recommended that each hospital uses their own diagnostic algorithm to reduce radiation and contrast exposure.

### Preoperative examinations

For conventional cardiac operations, risk scores such as the Society of Thoracic Surgeons Predicted Risk of Mortality (STS-PROM) [55] or EuroSCORE I and II [56] are well established; however, these scores have not been validated for open or endovascular treatment of aortic diseases [57].

These scores do not consider the frailty of older patients [51, 58–60]. These scores’ goal should be to provide a validated, standardized preoperative check of all vascular and cardiac comorbidities that can influence the outcome. The patients’ medical histories should be checked for ischemic heart disease [61], prior coronary stent implantation [62], heart failure [63], arrhythmias [63], heart valve disease [64] and arterial or pulmonary hypertension [65, 66].

Typically, for all patients with acute chest pain, ECG, chest X‑ray and transthoracic echocardiography (TTE) are used to exclude other diseases such as heart attack.

The ESC guidelines [43] recommend using TTE (class I, level C) as an initial imaging modality in all suspected cases of aortic dissection because of TTE’s availability. In Germany, TTE should be available in all certified chest pain units.

For definite diagnosis, transesophageal echocardiography (TEE), ECG-gated CT scans or MRI have the same reliability as one another for verifying an aortic dissection [67]. The distal part of the ascending aorta and the arch cannot be examined by TEE; however, TEE has the advantage of distinguishing between false and true lumens and visualizing entries using a color Doppler.

ECG-gated CT angiography of the whole aorta remains the gold standard for diagnosing aortic dissection. It is the most used imaging modality and can also visualize complications of side branches. The usage of ECG gating reduces motion artefacts at the level of the aortic root [68]. Precise planning for thoracic endovascular aortic repair (TEVAR) is possible when using multiplanar reformations [69]. Therefore, isotropic voxels should be generated.

For TEVAR planning, in addition to the whole aorta, access via iliac and femoral arteries should also be included in the scan. It is also beneficial to include supra-aortic vessels to assess the anatomy of vertebral arteries [68, 69].

### Intraoperative imaging

TEE can also be used in the intraoperative setting to evaluate wires and devices in the appropriate lumina [70].

Intravascular ultrasound (IVUS) is an important imaging modality, especially in TEVAR, for adequately sizing the aortic diameter of the landing zones, distinguishing between false and true lumens and diagnosing a true lumen collapse. Additionally, in emergency situations, where no appropriate CT scan is available, IVUS can be helpful [68, 71–73].

In these cases, IVUS-guided correctly sized stent graft diameters have a positive effect on remodelling and reduction of reintervention [73–75].

The usage of IVUS is recommended for sizing landing zones, visualizing side branches and reducing contrast.

### Postoperative imaging

Independent of treatment, approximately 60% of all patients with an aortic dissection present with aortic diameter progression [7].

CT controls (alternatively MRI, especially in younger patients) should be performed 30 days, 3, 6 and 12 months after TEVAR and, thereafter, annually and lifelong [8, 69]. If the CT shows a morphological change with no direct therapeutic consequence, the next CT control should be performed after 3 months.

After 5 years of stable results, controls can be performed in 18–24-month intervals. The extension of the dissection, thrombosis of the lumina, remodelling, perfusion of the false lumen and progression or regression should be noted [69].

**Fig. 5:**
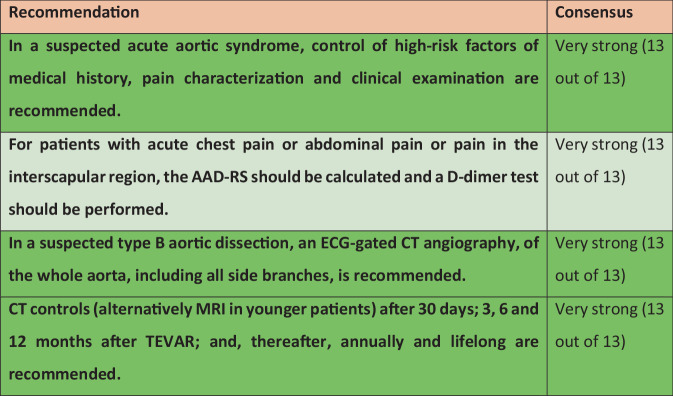
Recommendation 2: diagnostic testing

The CT scan should be performed in the native, arterial and venous phases [68, 76]. Late phases are important for visualizing the perfusion of the false lumen [76]. After endovascular treatment, endoleaks, positive regression of the aortic diameter, negative remodelling and progression of the aortic diameter should be noted [77]. Of special importance are complications, such as bird beak, distal stent graft-induced new entries (dSINE), occlusion of side branches and retrograde type A dissections [78–80]. The dSINE typically occur between 12 and 36 months after TEVAR, rarely occurring in the early phase (≤ 30 days) [8] and typically presenting as asymptomatic ([79, 81]; Fig. 5).

## Treatment

### Acute uncomplicated type B aortic dissection

#### International guidelines and expert consensus documents

Since the last publication of these guidelines no new recommendations or international guidelines have been published. During this period, the American Society of Vascular Surgery (SVS) only commented on aortic dissection in the TEVAR guidelines for aortic aneurysms.

Most existing guidelines are more than 10 years old (AHA, [6], Fattori et al. [7], ESC [43], Japanese guidelines [82]); the most recent guidelines were published by the European Society for Vascular Surgery (ESVS). The SVS, in collaboration with the American Society of Thoracic Surgeons (STS), recently published reporting standards but these standards omitted recommendations for treatment [8].

In 2021, the European Society of Cardiology (ESC) and the European Association of Cardiothoracic Surgeons (EACTS) published a position paper on the use of TEVAR in acute and chronic aortic disease [77]. This paper included the following recommendations:

- The recommendations for TEVAR in uncomplicated acute type B dissections include a primary entry tear > 10 mm, primary entry at the inner curvature, maximum total diameter > 40 mm and maximal diameter of the false lumen > 25 mm. Treatment should be performed in the chronic phase (15–90 days). Centralization of aortic treatment is recommended. One risk factor for treatment is the presence of a short distance from the primary entry to the left subclavian artery (LSA).

The clinical practice guidelines of the ESVS [83] recommend the following:

- To prevent late complications, an early TEVAR in selected cases can be taken into account (class IIb/level B).

The ESVS, in cooperation with the EACTS, published new guidelines about aortic arch pathologies [84]; however, the guidelines are only tangentially related to type B dissections.

#### Best medical treatment (BMT)

All patients with suspected or verified type B aortic dissection should be referred to a center that is specialized in diagnosing and treating aortic dissections and has all relevant specialized treatment options available [2, 43, 77, 84–87]. These specialist centers should have experience in open, endovascular and conservative treatment of aortic dissection. All patients with verified acute aortic dissection should be transferred to a monitoring station where hemodynamics and urine production can be monitored.

The goal of medical treatment is preventing rupture or deterioration of the dissection with consecutive deterioration of organ perfusion. This goal is reached by controlling blood pressure and heart rate and reducing the force of left ventricular ejection (dp/dt). Blood pressure between 100 and 120 mm Hg and a heart rate below 60 bpm is tolerable [86]. First-line treatment includes intravenous beta-blockers [86, 88]. If this treatment is not sufficient or the patient demonstrates intolerance to beta-blockers (asthma, bradycardia or impending heart failure), calcium channel blockers are indicated, which also reduce the risk of reflex tachycardia. Esmolol would be the first recommended choice of blocker, due to its short half-life; alternatively, metoprolol or labetalol are also recommended [86].

In large trials, improved survival and decreased aortic expansion rates were found for the administration of beta-blockers and calcium channel blockers [88–91]. In patients with inadequate blood pressure control, vasodilators are the first alternative choice. These drugs should not be administered as monotherapy because of the increased wall shear stress [86].

After IV treatment, the next goal is to transition to oral medication. In this case, beta-blockers and calcium channel blockers are also the first-line treatment, followed by renin-angiotensin system inhibitors or alpha1 receptor blockers [85]. The limits of blood pressure must be raised in cases of organ dysfunction (e.g. renal failure) [6, 43].

For all patients who are in monitoring units and receive IV medication, blood pressure should be monitored through invasive measurements; this monitoring should continue until systolic blood pressure and heart rate control are gained and no organ dysfunction is observed.

In addition to controlling blood pressure, it is also important to treat pain. Pain can increase blood pressure and therefore deteriorate the aortic dissection. In this case, IV treatment and opiates are the first-line treatment. Refractory pain is an alert signal and can indicate progression or impending rupture [92].

In the chronic phase, blood pressure < 130/80 mm Hg should be targeted. Additionally, statin treatment should be initiated [85, 93].

There is little research addressing the recommended duration of intensive care monitoring. In a retrospective study from Japan, 73 consecutive patients with uncomplicated type B aortic dissections were investigated [94]. The first 39 patients were treated by following traditional protocols published in the Japanese guidelines [82]. These include a relatively long time of bedrest and cautious mobilization at day 7. All patients treated after 2013 (*n* = 34) received a fast-track program. For these patients, the amount of rest was reduced: for example, on day 2, patients sat on the bed; on day 3, patients stood by the bed. All actions were undertaken with blood pressure monitoring. The complication rate was reduced in the second group. Adverse aortic events did not (significantly) occur in the fast-track group. In this group, rates of pneumonia, duration of IV treatment (3.8 ± 1.4 vs. 3.0 ± 1.4 days), length of hospital stay and, therefore, total costs were significantly reduced. Additionally, delirium was reduced but not significantly.

In a 2009 trial following a similar fast-track program, the results were similar; however, a higher number of patients were included (90 standard protocol vs. 120 with fast-track protocol) [95].

The optimal duration of intensive care monitoring is unclear; in these two studies, no robust data that would provide clear recommendations were presented. The authors suggest that patients remain stable under oral medication and that imaging also remains stable.

Durham et al. [96] reported long-term results after primary conservative treatment of acute aortic dissection. From 298 patients, 200 received long-term follow-up with available imaging. After a median follow-up of 4.3 ± 3.5 years, 119 patients (39.9%) died. Aorta-related interventions occurred in 87 (29.2%) of the patients, predominantly (56 patients) due to aneurysmal degeneration. The median growth rate was 12.3 mm/year for the total diameter and 3.8 mm for the false lumen.

In a systematic review [97] of 15 studies and 2347 patients, the 30-day mortality was 2.4%, the pooled cerebrovascular incidence was 1% and the rate of spinal ischemia was 0.8%. The 1‑year survival rates were 86.2–100%, and, after 5 years, the survival rates were 59–97.2%. Freedom from aortic events after 5 years was 34–83.9%.

Interestingly, the 5‑year mortality rates in Japan were lower (12.8–13.2%) [98]. The reasons for this discrepancy are unclear; recommendations on medical treatment remained similar [82].

It is currently unclear if a primary conservative approach with best medical treatment should be the treatment of choice in non-A-non‑B dissections. In a meta-analysis [99] including 433 patients, the 30-day mortality was 14%, which is clearly higher than in type B dissections. For patients who were treated by an open, hybrid or endovascular approach, the 30-day mortality was 3.6%, the rate of retrograde type A dissections was 2.6% and the stroke rate was 2.8%.

#### Endovascular treatment

Only one randomized multicentric trial exists that compares BMT with TEVAR; this research was industry-sponsored. The primary endpoint of the acute dissection stent grafting or best medical treatment (ADSORB) trial was aortic remodelling after 14 days of TEVAR [100]. In the BMT group, 31 patients were recruited, whereas in the TEVAR + BMT group, 30 patients were included. Three patients from the BMT group received TEVAR due to expansion of the aorta; after 1 year of randomization, one malperfusion and two aneurysms were noted. In the TEVAR + BMT group, one patient died. An incomplete thrombosis was found in 97% of the BMT group and in 43% of the TEVAR + BMT group. Regression of the diameter of the false lumen and an increase of the true lumen were only found in the TEVAR + BMT group. The total diameter was constant in the BMT group but reduced in the TEVAR + BMT group from 42.1 to 38.8 mm (*p* = 0.062). Long-term data are missing from this study.

Xie et al. [101] analyzed the outcome of 267 TEVARs that were administered in the acute or subacute phase with a median follow-up of 48.2 ± 25.9 months. Indication was seen in expanded criteria (40 mm initial diameter, false lumen diameter of 22 mm, primary entry > 10 mm and partial thrombosis of the false lumen). No significant difference was found regarding mortality, spinal ischemia and stroke, although mortality was fivefold higher in the acute phase (five patients vs. one patient). Aortic rupture, retrograde type A dissection and major stroke only occurred in the acute phase; 90% of all patients had a complete thrombosis of the false lumen in the thoracic aorta and 30% had a stable diameter in the abdominal aorta. Freedom from aorta-related complications was between 84% and 90% after 5 years.

In a single center analysis, [102] all patients with uncomplicated type B dissections treated over a 12-year period were investigated (751 patients). The in-hospital mortality rate was 0.7% and long-term mortality was 52.8 ± 10.9 months. The 10-year survival rate was 83%, and the reintervention rate was 7.9%. Retrograde type A dissections occurred in 0.5% of cases.

The optimal time for endovascular treatment is still under debate. Whereas some authors [101] have not found a difference between the acute, subacute and chronic phases, other trials [100, 103] found a higher complication rate in the acute phase and a lower complication rate in the long term. The TEVAR data in acute and subacute settings are shown in Table 4.

**Table 4:** Complication rate after TEVAR in the acute and non-acute phase

	Acute [103–106]	Non-acute [103, 107, 108]
30-day mortality	0.5–7.1%	0–4.5%
Retrograde type A dissection	0.5–1.6%	0–1.5%
Perioperative stroke	0.5–6%	0–1.5%
Spinal ischemia	0–3.4%	2.9–4.5%

#### Indication and comparison of treatment modalities

A 2020 meta-analysis [109] investigated 14,706 patients. Only 6 studies were included; all other studies (325) were excluded because of a lack of comparison group data. Patients treated with TEVAR had a higher rate of perioperative strokes, but BMT resulted in higher long-term mortality (all-cause and aorta-related). All other parameters (e.g. in-hospital mortality) presented no difference. The indication for treatment was not investigated. These criteria may be able to predict late aortic events. Patients with these criteria are at high risk and some authors assessed these patients against a group of extended criteria for complicated type B aortic dissections [8, 108, 110].

A meta-analysis from 2018 [111] evaluated these criteria by investigating a number of groups over a period of 10 years. In total, 51 studies with 8074 patients were included. Only one negative predictor could be found:

- Maximal total aortic diameter at presentation > 40 mm.

All other predictors were considered to be controversial because most of the included trials were single-center analyses with low numbers of patients (Table 5).

**Table 5:** High-risk criteria of uncomplicated acute type B aortic dissection

	Evidence class	Evidence level	Reference
Maximal total aortic diameter at presentation > 40 mm	IIA	B	[17, 96, 112–128]
Complete thrombosis of the false lumen	IIA	B	[118, 129]
Primary entry tear > 10 mm	IIB	C	[108, 130]
Partial thrombosis of the false lumen	IIB	C	[117, 118, 131, 132]
Primary entry tear at the inner curvature	IIB	C	[133–136]
Maximal false lumen diameter at presentation > 22 mm	IIB	C	[117, 120, 127, 130, 135–139]
Ratio of true/false lumen < 0.8	IIB	C	[137]
Ulcer-like projections	IIB	C	[113, 140]
Fibrinogen-fibrin degradation products > 20 mg/ml	IIB	C	[123]
Number of involved side branches	IIB	C	[112, 120, 135, 139, 141]

The meta-analysis discussed ulcer-like projections in-depth, especially in patients with IMH. The meta-analysis only expressed a IIB recommendation. In 2021, a new study was published [140]; within 5 years, 62 patients with ulcer-like projections were retrospectively compared with 78 patients without ulcer-like projections. The follow-up occurred after 1 year. High-risk patients presented with ulcers of a depth of 5 mm or situated in the proximal aorta. These factors were independent risk factors of late aortic events.

Newer studies have investigated additional predictors for late aortic events, like the distance of the primary entry to the LSA and ejection fraction of the false lumen, which are measured with 4D MRI [142]. Only 18 patients were included, and 4D MRI was only available in selected centers; additionally, 4D MRI exams can last for as long as 60 min [78].

A new approach is to combine predictors into a score model. Matsushita et al. [143] analyzed 187 consecutive patients from 2 centers. After they established their model, it was validated with 219 patients in 4 Japanese centers (Table 6).

**Table 6:** Score model of predictors for late aortic events

Maximal total aortic diameter at presentation > 40 mm:	2 points
False lumen lager than the true lumen:	2 points
Ulcer-like projections:	1 point
Age ≥ 70 years:	1 point

Patients with a score of 2–6 had a significantly higher risk for late aortic events.

**Fig. 6:**
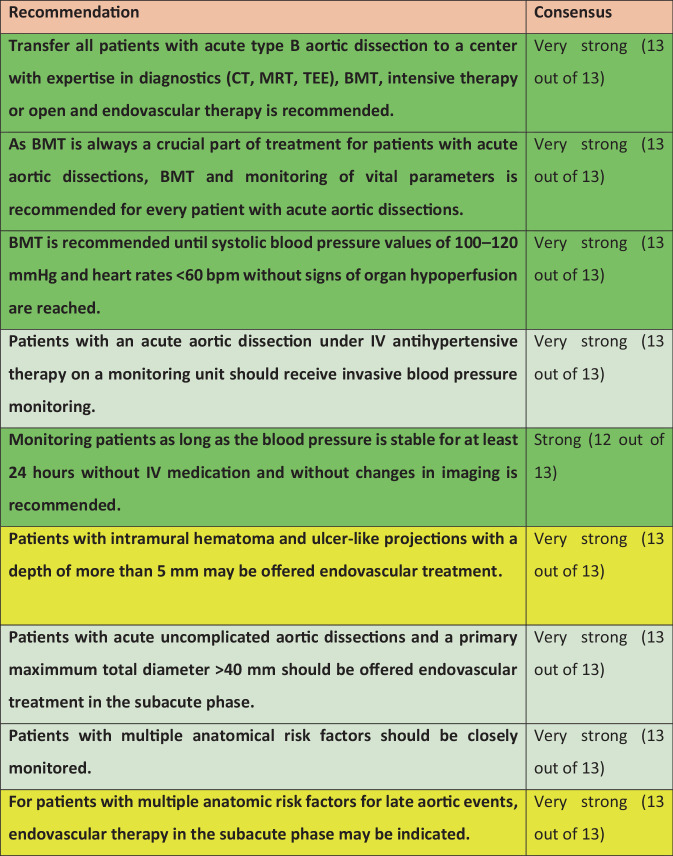
Recommendation 3: treatment of acute uncomplicated aortic dissections

Sailer et al. [144] investigated the importance of aortic remodelling. If the diameter increases by 5 mm in the first 6 months, there is a 2.29-fold increased risk for late aortic events compared to patients that have an enlargement of 2.4 mm over the same period. Similar results were found in another single-center analysis [145]. Patients with an enlargement of 2–5 mm in the first 2 weeks had a similar risk for late aortic events as patients with a primary diameter of 40 mm (3.64 vs. 2.96) (Fig. 6).

### Acute uncomplicated type B aortic dissection

Complicated type B aortic dissections, are defined as type B dissections with: [7, 8]

- rupture
- malperfusion with
  - o ischemia of visceral organs
  - o leg ischemia
  - o spinal ischemia
- high-risk patients with
  - o refractory pain
  - o refractory hypertension
  - o rapid expansion of the aortic diameter

The outcomes of patients with complicated dissections are significantly worse than those with uncomplicated dissections. In a single-center analysis of 442 patients, the in-hospital mortality was 16.1% vs. 2.6% [146].

#### TEVAR vs. open repair

TEVAR is recommended as the first-line treatment with class I, level C evidence in the current ESC guidelines [43]. There have not yet been any randomized trials comparing endovascular and open repair in acute complicated type B dissections. The ESVS guidelines have similar recommendations: [42]

- Patients with acute complicated type B dissections should receive TEVAR as the first-line therapy (class I/level C).
- Open repair is an alternative treatment if TEVAR is not feasible or fails (class IIA/level C).
- For malperfusion patients, endovascular fenestration is an option (class IIA/level C).

In Moulakakis et al. [97] meta-analysis of 30 studies, 2531 patients with acute complicated type B dissections treated by TEVAR the pooled in-hospital mortality rate was 7.3%. The pooled cerebrovascular event rate was 3.9%, and the spinal ischemia rate was 3.1%. The 1‑year survival rate varied between 62% and 100%; the 5‑year survival rate varied between 61% and 87%. Freedom from late aortic events after 5 years was 45–77%. A meta-analysis of open repairs including 9 studies and 1276 patients found worse results. In this meta-analysis, the pooled cerebrovascular event rate was 6.8%, the spinal ischemia rate was 3.3% and the cumulative rate of unwanted neurological events was 9.8%. The 1‑year survival rate varied between 74.1% and 86%, and the 5‑year survival rate varied between 44% and 82.6%. Data to calculate the freedom from late aortic events were missing.

An additional systematic review found a 30-day mortality after TEVAR (in 1574 patients) of 8.07% and a morbidity rate of 30.8% [147].

A 2014 systematic review [148] including 134 patients with peripheral malperfusion and leg ischemia reported that 22% of patients received conservative treatment. In patients with open repair (fenestration and extra-anatomic bypasses), the 30-day morbidity was 31% and the 30-day mortality 14%. In the endovascular group (50% fenestration, 32% TEVAR), the results were 46% and 8%, respectively. In a recent single-center analysis with a higher rate of TEVAR, the in-hospital mortality was 7%. The group that received primary and peripheral intervention experienced a higher failure rate than the group with primary TEVAR.

A prospective trial [149] included 50 patients over 5 years. The 5‑year freedom from dissection-related mortality and secondary interventions was 83% and 85%, respectively. After 5 years, 94% presented with complete remodelling and a stable true lumen of the thoracic aorta.

Several analyses of national databases [87, 150–152] exist with high patient numbers. However, all of these analyses suffer from an inability to distinguish between TEVAR in complicated dissections and uncomplicated dissections. Additionally, discriminating between type A and type B dissection is not always possible in patients who received open repair. The National Inpatient Sample [87] of the USA reported that 1540 patients with type B dissections had an in-hospital mortality rate of 15.3% after open repair and 4.9% after TEVAR (1130 patients) in 2012.

In a national database from Taiwan that included 1542 OR patients and 119 TEVAR patients (no information about complicated or uncomplicated), the 30-day mortality was 4.2% after TEVAR and 17.8% after open repair. After 4 years, 68% of patients after open repair and 79% after TEVAR were still alive [151].

Luebke und Brunkwall [153] state in their meta-analysis that TEVAR has a significantly lower mortality and paraplegia rate with no significant differences in the long term. Based on these data, they calculated the cost-effectiveness [154] of both treatment strategies. Open repair was more expensive and less effective in terms of quality-adjusted life years (QALYs).

Hogendoorn et al. [155] published similar results, although the reintervention rate was higher in the TEVAR group than in the open repair group. TEVAR was more effective in all age groups.

Although existing meta-analysis and multicenter analysis included a higher number of patients, most analyses did not distinguish between complicated and uncomplicated type B aortic dissections.

There is no consensus on the minimal recommended length of the stent graft. However, what is clear is that shorter stent grafts have a higher reintervention rate [156] and, by covering one third of the thoracic aorta, further expansion can be stopped [157]. The reintervention rate correlates in one multicenter retrospective study (814 patients in 15 years) with the length of the stent graft. Best results were achieved with extension to the celiac trunk. Unfortunately, no spinal ischemia rate was given in the study.

#### Special techniques

##### Fenestration.

During fenestration, a connection between true and false lumen is made. This can be performed by using an endovascular or an open approach. The aim is to treat malperfusion due to compression of the true lumen.

Trimarchi et al. [158] study presented the longest follow-up after open aortic fenestration. In total, 18 patients were treated, 16 as emergency and 2 within 48 h, who presented with refractory hypertension and refractory pain. The fenestration was made in 10 patients in the suprarenal and 8 patients in the infrarenal part of the aorta. The in-hospital mortality was 22% (84 patients); during the 10-year follow-up, 3 other patients died of non-dissection related causes. In the remaining 11 patients, the result was satisfactory, no other ischemic complication occurred and no reintervention was necessary. In the treated segment, no dilatation was noted, but five patients developed aneurysms in other segments.

In a larger case series, 42 patients were open fenestrated in the suprarenal region [159]. The 30-day mortality rate was 21.4% and the 5‑year mortality rate was 70.6%. In the follow-up, five patients died of top aortic rupture. The indication for operation was malperfusion in only 17 patients. All other patients were operated on because of refractory pain or rapid progression.

The largest case series of endovascular fenestrations was published by Norton et al. [160]. Between 1996 and 2018, 99 patients with malperfusion were treated by fenestration with or without additional stent. The 30-day mortality rate was 7.7%, and in the last 8 years of follow-up no patient died. The 5‑year and 10-year survival rates were 72% and 49% and the reintervention rates were 21% and 31%, respectively.

##### PETTICOAT.

PETTICOAT refers to the provisional extension to induce complete attachment [161]. This technique compares an occlusion of the primary entry tear with a covered stent and distal extension with an uncovered stent. The 5‑year results of the STABLE I trial (Study of Thoracic Aortic Type B Dissection Using Endoluminal Repair) [162] and 1‑year results of the STABLE II trial [163] were published. Both were non-randomized multicenter trials. In the feasibility trial (STABLE I), acute and subacute complicated type B dissections were included, whereas in the STABLE II only acute complicated aortic dissections were included. In STABLE I, 55 patients with acute type B dissections were treated. The 30-day mortality rate was 5.5%, the 5‑year survival rate was 79.9 ± 6.2% and the aneurysm-related survival rate was 83.9 ± 5.9%. A complete thrombosis of the false lumen was achieved in 74.1% of the cases. In STABLE II, 20 patients with rupture and 57 with malperfusion were included. The 30-day mortality rate was 6.8% and the 1‑year survival rate was 80.3%. The periprocedural stroke rate was 6.8% and the rate of spinal ischemia was 5.5%. A complete remodelling occurred in 78.3% of cases.

In a comparison of TEVAR (841 patients) and PETTICOAT (84 patients from the STABLE II trial), the 30-day mortality rate was 17.1% vs. 8.3% (not significant), respectively [164]. The malperfusion-associated 30-day mortality was significantly lower (12% vs. 2.4%; *p* = 0.038). The diameter of the true lumen in the abdominal part was significant larger in the PETTICOAT group.

A meta-analysis [165] and a Cochrane review [166] stated that no clear statement can be given due to the heterogeneity of the trials. These studies stated that re-expansion of the true lumen can only be improved in the short term.

##### STABILISE.

A further development of the PETTICOAT principle is the Stent Assisted Balloon Induced Intimal Disruption and Relamination in Aortic Dissection Repair (STABILISE). In addition, in the PETTICOAT procedure, the stent graft in the thoracic aorta is dilated with a compliant balloon to disrupt the intima and seal the stent graft. Two groups published their data. In the Italian group [167] only acute complicated patients (*n* = 10) were included. The overall mortality after 7.2 months was 0%. In all patients, a complete expansion of the true lumen was achieved and the abdominal part of the aorta remained stable.

In the French group [168] 41 patients with complicated type B aortic dissection or a primary diameter > 40 mm were included. The 30-day mortality rate was 2%, and, after a mean follow-up of 1 year, 20% of patients required reintervention. The false lumen was completely thrombosed in the thoracoabdominal aorta. Below the stent, the false lumen was thrombosed in only 39% of cases; 5% of cases presented an increase in diameter.

Currently, a multicenter register is in process, but no data have yet been published by this register (clinicaltrials.gov NCT03707743).

##### Bare metal stent implantation.

Examples of small diameter bare metal stent implantation in the true lumen in cases of true lumen collapse are very limited. Besides case reports, only 1 single-center study [169] with 14 patients has been published. The study’s patients received stents with diameters ranging from 7 to 28 mm. For one patient, additional open revascularization was necessary, and four patients required additional fenestration. During the first week, four patients experienced a collapse of the implanted stents; three of these patients could be recanalized by performing PTA. No middle-term data were included in the study, and the effects of further endovascular treatment in these patients with small diameter stents is unclear.

##### Total hybrid arch repair—Frozen elephant trunk.

In selected cases, in particular if no adequate landing zone is available, the frozen elephant trunk technique may be required. The prevalence of experience with this technique is low and the results have been found to be inconsistent. In a single-center analysis [170] of 21 patients, the perioperative mortality, spinal ischemia and stroke rates were 3%, 0% and 14%, respectively; in a multinational register [171] of 57 patients, these rates were 14%, 4% und 10%, respectively. In both case series, a significant number of patients required subsequent distal extension.

In a study with a larger cohort [172] of 79 patients, the perioperative mortality, spinal ischemia and stroke rates were 5.1%, 3.8% and 2.5%, respectively. The freedom from distal interventions was 97.3%, 97.3%, 87.8%, 84.3% and 79.3% after 6 months and 1, 3, 5 and 7 years, respectively.

#### Hemodynamic monitoring

**Fig. 7:**
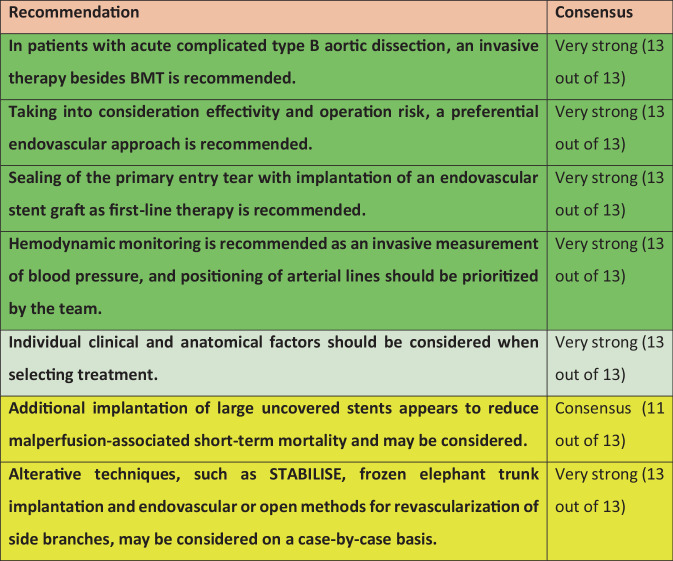
Recommendation 4: treatment of acute complicated aortic dissections

The hemodynamic monitoring depends on the type of anesthesia given to patients and the experience of the center. For standard monitoring in all operations and interventions, an invasive blood pressure measurement should be performed proximal to the dissection. Ideally, consent on all possible accesses should be found between interventionalists, surgeons and anesthesiologists. For renal perfusion and renal output control, a transurethral catheter is reasonable. For monitoring cerebral perfusion, near-infrared spectroscopy (NIRS) is possible, especially in cases where the aortic arch is involved (Fig. 7).

### Subacute type B aortic dissection

The subacute phase is defined as the time between 15 and 90 days after the primary event. There is only one randomized study that addresses the subacute phase. In INvestigation of STEnt grafts in patients with type B Aortic Dissections (INSTEAD) [173], BMT (*n* = 68) and BMT+TEVAR (*n* = 72) uncomplicated type B dissections were evaluated. The patients were randomized after presenting with uncomplicated aortic dissections and completing an uneventful acute phase. After 1 year, there was no significant difference between the two groups regarding overall mortality, aorta-related mortality or aortic progression [174]. However, the complication rate was higher in the BMT+TEVAR group. Additionally, the aortic remodelling was significantly higher in the BMT+TEVAR group. After extension (INSTEAD-XL), the 5‑year data were made available [108]. Aneurysm-related mortality and diameter progression were significant lower in the BMT+TEVAR group than the BMT group. Additionally, the overall mortality was lower but not to a significant degree. The false lumen thrombosis was 90.6% in the TEVAR group.

In most single-center analyses, indications were found because of extended criteria for complicated type B dissections (> 40 mm total diameter, > 22 mm false lumen diameter or > 10 mm primary entry) [101] and signs of progression or instability (increase of diameter > 4 mm, new para-aortic hematoma or hemorrhagic pleural effusion) [7].

In a comparative single-center study, [101] the outcomes of 267 TEVARs in the acute or subacute phase were analyzed.

**Fig. 8:**
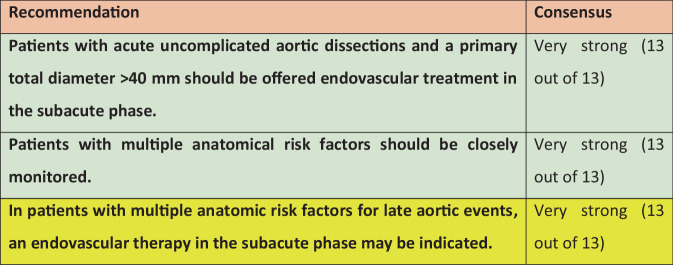
Recommendation 5: treatment of subacute aortic dissections

The impact of TEVARs on blood pressure was analyzed by Usai et al. [175]; reduction of blood pressure was significantly higher in the TEVAR group than in the BMT group. This effect was pronounced in the group with refractory hypertension (≥ 5 antihypertensive medications) (Fig. 8).

### Chronic type B aortic dissection

The chronic phase begins 90 days after the primary presentation. A major problem in this phrase is progression to a postdissection aneurysm. For this reason, lifelong surveillance is important [8]. Indication for treatment has been defined in several consensus documents as the following: [7, 176]

- Total diameter 55 mm
- Rapid progress of more than 4 mm [7] or 10 mm [176] in 1 year
- Repeated chest or back pain with no other cause
- Refractory hypertension despite maximal antihypertensive medication, in conjunction with small true lumen and renal malperfusion
- Rupture
- Malperfusion

A recent systematic review [177] compared the differences between the existing guidelines (Table 7).

**Table 7:** Overview of recommendations of the different guidelines in chronic dissections

	ACCF/AHA 2010 [6]	JCS 2011 [82]	ESC 2014 [43]	ESVS 2018 [83]	SVS 2020 [69]
Maximal diameter of the thoracoabdominal aorta	IB (> 55 mm)	IC (> 60 mm)	IC (> 60 mm)	IIa (> 55 mm)	No information available
Open repair in symptomatic patients or aneurysmatic aortic dissection with low operative risk	IB	No information available	IC	IIa C	No information available
Endovascular repair symptomatic patients or aneurysmatic aortic dissection with medium/high operative risk	IB	No information available	IC	IIa C	No information available

#### Open repair

For the past few decades, open repair was considered the only possibility for treating chronic dissections. Since 2000, however, endovascular repairs have rapidly increased. One systematic review [178] included data from all larger studies (1574 patients). The authors of the review divided the studies into pre-endovascular and post-endovascular eras (Table 8).

**Table 8:** Morbidity and mortality after open repair of chronic type B aortic dissection

	Pre-endovascular era (%)	Post-endovascular era (%)
In-hospital mortality	15.2	7.5
1‑year survival	82.1	
3‑year survival	74.1	
5‑year survival	66.3	
10-year survival	50.8	
Stroke	5.3	5.9
Spinal ischemia	4.6	5.1
Acute renal injury	13.5	8.1
Late aortic intervention	13.3	11.3

The study included centers with more than 100 patients and hospitals with only 12 patients in the same period. The operation techniques also significantly differed between centers and hospitals (hypothermia with cardiac arrest, left heart bypass, etc.).

##### Hemodynamic monitoring.

Hemodynamic monitoring of patients with type B aortic dissections depends on the type of anesthesia administered and type of operation undertaken. Left heart bypass, distal perfusion with extracorporeal membrane oxygenation and hypothermia with cardiac arrest are possible methods that differ between centers.

In the standard thoracoabdominal approach, the patient is positioned in a lateral right decubitus position. Typically, a distal aortic and, in selected cases, a selective visceral perfusion are used. In all cases, separate invasive blood pressure monitoring of the upper and lower parts of the body is indicated. Measuring the right radial and right femoral artery is standard; alternative approaches require an interdisciplinary agreement between the surgeon, anesthesiologist and perfusionist. For cardiac monitoring, a TEE or Swan-Ganz catheter is beneficial. For exposure of the thoracic aorta, single-lung ventilation, which can be achieved by using a double-lumen tube or bronchial blocker, is required.

In cases of permissive hypothermia, a thermal probe is placed in the nasopharynx and in the rectum. For additional cerebroprotection, the administration of external cool packs to the head is beneficial. Large-bore peripheral intravenous lines and central venous lines in addition to a transurethral urine bladder catheter are obligatory.

#### Endovascular treatment

In one systematic review [179] midterm results up to 5 years after TEVAR were analyzed. In total, 16 studies with 567 patients were included. Technical success was defined as complete occlusion of the primary entry with absence of type I and type III endoleaks and no conversion to open repair. The 30-day mortality was 3.2% and the technical success rate was 89.9% with a median follow-up of 26.1 months. The overall mortality was 9.2%. In the late follow-up 7.9% of the patients developed an aneurysm in the distal part of the aorta with persistent perfusion of the false lumen.

In another review [180] the median reintervention rate was 18% (28 studies, 1249 patients and a median follow-up of 27 months). In 3.9% of the cases, a conversion to open repair was necessary. The late overall mortality rate was 9.9% (3.6 deaths per 100 patient years), the aorta-related mortality 3.9% (1.1 deaths per 100 patient years) and reintervention mortality 3.1% (3.6 deaths per 100 patient years). The rupture rate was 0.7 per 100 patient years, whereas 47.6 of the ruptures were due to distal reperfusion of the false lumen. The main cause of reintervention was aneurysmatic degeneration of the aorta at the distal end of the stent graft. In the review, the reintervention rate was high, but most of the reinterventions were endovascular procedures with low peri-interventional mortality rates.

In contrast to an open repair with TEVAR, in which only the primary entry is occluded, the false lumen is normally perfused via distal re-entry. The rate of a complete remodelling of the thoracoabdominal aorta in the chronic phase is low. Staged reinterventions are, in many centers, part of the treatment strategy for chronic dissections to minimize the spinal ischemia rate [181]. There are different strategies to minimize the high rate of reinterventions.

##### Hybrid.

Only a few publications exist that address combining the endovascular approach for treating the thoracic part of the aorta with an open repair of the abdominal aorta. In single-center analyses [182–184] concerning this combined approach, the mortality rate was 0–7%, stroke rate 0%, acute renal failure rate 20–26% and paraplegia rate 0–5% in up to 31 patients. Wang et al. [181] published a study that used the same approach but applied the approach to degenerative thoracoabdominal aneurysms. Long-term results are missing. This staged hybrid approach is also feasible as a 3-stage procedure with a prior frozen elephant trunk [185].

##### Fenestrated/branched stent grafts.

Additionally, this study uses data from single-center analyses with low patient numbers. Kitagawa et al. [186] previously published data concerning 15 patients in 2013. In their study, the peri-interventional morbidity, mortality, spinal ischemia, acute renal failure and stroke rates were 0%. Furthermore, the reintervention rate was low.

The largest cohort studies were published in 2019 by two centers in Germany [187]. These studies included 79 patients. In-hospital mortality was 5.6%, and the spinal ischemia rate was 4.3%. The 1‑year, 2‑year and 3‑year survival rates were 84.7 ± 4.5%, 80.7 ± 5.3% and 70 ± 6.7%, respectively. Freedom from reintervention was 80.7 ± 5.3%, 63 ± 6.9% and 52.6 ± 8%, respectively.

##### False lumen occlusion.

In aortic rupture, covering the primary entry is not always sufficient for treatment because of the potential for reperfusion of the false lumen via type R endoleaks [8]. To overcome this problem, the concept of false lumen occlusion was developed. False lumina can be addressed by using the Knickerbocker technique, the STABILISE technique, plug coils, Onyx glue or dedicated occlusion devices (e.g. the candy-plug technique). A 2019 systematic review compared these techniques [188]. A total of 61 patients were included. The 30-day mortality rate was 0%, and the technical success rate was 99%, but the false lumen thrombosed in only 62% of the patients. No patient died until the end of the follow-up.

### Comparison of treatment

A systematic review from 2019 [189] compared open repair with TEVAR for chronic type B aortic dissections. The review included 39 studies (including 4 comparative studies) with a total number of 1079 patients in the open repair group and 1271 patients in the TEVAR group. The endovascular approach, compared to the open repair approach, presented significantly lower perioperative mortality (9.3% vs. 2%), stroke rates (4.5% vs. 2.7%), spinal ischemia (5% vs. 2.7%) and dialysis (5.2% vs. 0%). The 1‑year and 3‑year mortality rates were comparable between the groups, but the reintervention rate was significantly higher in the endovascular group (14.7% vs. 34%). Reinterventions in the OR group were predominantly open repair and in the TEVAR group endovascular interventions. Late aortic rupture occurred in 1.2% (OR group) and 3% (TEVAR group) of cases. Remarkably, most interventions in the endovascular group were TEVAR, and only two studies included fenestrated branches or stent grafts.

### Distal stent graft-induced new entry (dSINE)

Occurrence of a new entry, mostly at the distal end of the stent graft, is a known problem that should not be underestimated. Since first publication in 2010 [190] two further systematic reviews [191, 192] addressing new entries have been published. D’Cruz et al.’s analysis [191] is the most extensive of the two. In 17 studies concerning 3962 patients, dSINE occurred in 7.9% of cases after a median follow-up of 19 ± 7 months. Risk factors included extensive distal oversizing of > 20% (odds ratio 2.06); short stent grafts (< 145 mm), in which the stent graft does not reach the straight part of the descending aorta; and, interestingly, treatment in the chronic phase (odds ratio 3.12). In contrast, a single-center analysis [193] with 187 patients found acute aortic dissections to be an independent predictor rather than chronic aortic dissections.

**Fig. 9:**
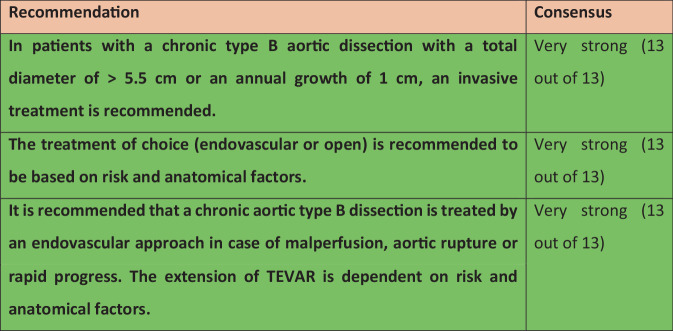
Recommendation 6: treatment of chronic aortic dissections

In a recent publication [194] short-term data concerning using a dedicated stent graft to prevent dSINE were published. In this context, the stent graft should be tapered and the last two struts removed. In 13 patients treated using this type of dedicated stent graft, only one patient developed a dSINE after a median follow-up of 17 months (Fig. 9).

## Spinal ischemia

Spinal ischemia is one of the major complications following treatment of thoracic and thoracoabdominal pathologies. Ischemia is induced by covering spinal arteries with stent grafts or by oversewing during an open repair. The α‑motor neurons in the anterior horn are especially vulnerable to ischemia [195]. After the ischemic phase, reperfusion injury is an important part of pathophysiology. In humans, the spinal cord is perfused by a collateral network. Subclavian arteries, hypogastric arteries, deep femoral arteries and epigastric arteries are significant parts of this network [196, 197].

Paraplegia and paraparesis with sphincter incontinence dramatically reduce the quality of life. The incidence of spinal ischemia after TEVAR was 3 7% [198] in the Vascular Quality Initiative, which included 11,473 procedures across 5 years. In this initiative, the 1‑year survival rate was significantly reduced (65%), compared with patients without spinal ischemia (87%). Mortality was also higher in patients with permanent paraplegia (84%) than in those with transient paraplegia (54%). Another study found similar results [199].

The incidence of spinal ischemia varied from < 1% to 20%, depending on the underlying pathology and treatment modality. Most data came from degenerative thoracoabdominal aneurysms. In a recent meta-analysis [200] the rate of spinal ischemia was 1.8% after TEVAR in aortic dissection patients, compared with 5.73% in degenerative aneurysm patients.

The rates of spinal ischemia in aortic dissections patients are shown in Table 9.

**Table 9:** Spinal ischema rates

	Conservative treatment	Open repair	Endovascular treatment
Acute uncomplicated type B aortic dissection	0.8% [97]	n.a.	0–4.5% [103–108]
Acute complicated type B aortic dissection	n.a.	3.3% [97]	3.1% [97]
Chronic type B aortic dissection	n.a.	5–9% [189, 201]	2.7% TEVAR [189] 8% FEVAR [201]

### Risk of spinal ischemia

Risk factors for spinal ischemia include the following [198, 202–205]:

- extensive aortic pathology/treatment (< 20 cm)
- pathologies of the thoracolumbar transition
- long clamping times in open repair
- previous aortic treatments (e.g. abdominal aortic repair)
- occlusion of collateral (e.g. hypogastric artery)
- chronic renal insufficiency
- perioperative hypotension
- female sex
- urgent repair
- COPD

Some of these risk factors can be influenced by a strict perioperative protocol, whereas other risk factors cannot be influenced [206]. Such a protocol is also recommended by the U.S. Aortic Research Consortium [207].

### Prevention of spinal ischemia

Currently, there is no authorized medicine for preventing spinal ischemia. Although a long list of drugs have been animal tested and used in clinics (mannitol, methylprednisolone, naloxone, erythropoietin, H2S, etc.), [6, 208] no therapeutic substance has gained wide acceptance or routine usage [207]. In the ACC/AHA 2010 guidelines [6] some of these drugs received a class IIB recommendation. Systematic reviews or meta-analyses have not been performed on the efficacy of these drugs.

The main barrier to making general recommendations is the low incidence of aortic dissections. However, certain recommendations for degenerative aortic diseases can be adapted to this context [209]:

- avoidance of hypotensive phases (MAP > 90 mm Hg)
- shortest possible treatment length of the aorta
- staged approach in endovascular procedures
- revascularization of the LSA
- preservation of the hypogastric arteries
- spinal drainage
- local or systemic hypothermia in open repair
- optimizing hemoglobin values
- neurophysiologic monitoring
- perioperative coiling of spinal arteries

Not all recommendations are realizable and there are relevant differences between open and endovascular repair. While some recommendations are clear (avoidance of hypotensive phases, treatment length, etc.), other points should be reviewed further for these guidelines.

#### Spinal drainage

The aim of spinal drainage is to reduce the elevated pressure on the spinal cord induced by ischemia and reperfusion injury and the consecutive edema. Especially in open surgery, the use of spinal drainage is well-established and included in several guidelines (ESVS, ACC/AHA, JCS) [6, 42, 82]. In endovascular interventions, spinal drainage is also recommended for high-risk interventions (ESVS, ACC/AHA, SVS, ESC) [6, 42, 43, 69].

A Cochrane review from 2012 [210] found only limited evidence supporting prophylactic spinal drainage in open repair but recommended this type of drainage as part of a multimodal approach.

A systematic review [211] concerning spinal drainage in endovascular procedures gave no recommendations because of lacking evidence; however, the incidence of spinal ischemia was low. In a database analysis of the Vascular Quality Initiative and the SVS, the rate of spinal ischemia in degenerative aneurysms decreased after TEVAR or complex TEVAR from 4.55% in 2014 to 1.43% in 2018.

The risks associated with spinal drainage cannot be ignored. In a large cohort of 187 patients with 240 preventive spinal drainages in endovascular procedures, 19 patients (10%) suffered from complications. For these patients, 17 out of 19 presented with moderate to severe complications and 4 patients (2%) experienced paraplegia due to the spinal drainage [212]. In this study, 30% of all spinal ischemias were induced by spinal drainage. In a larger but not recent study [213] of a total of 486 patients treated by open repair, the complication rate was 6.5%, including 6 patients with post-puncture headaches, 24 patients with bloody spinal fluid (including fatal cerebral hemorrhage) and 2 patients with neurologic symptoms.

In a single-center analysis [214] of 473 spinal drainages after open and endovascular procedures over 4 years, the complication rate was 27%, including 2.3% of cases presenting with cerebral hemorrhages.

#### Neuromonitoring

The standard neurophysiological monitoring techniques are motor (MEPs) and somatosensory evoked potentials (SEPs). Whereas SEPs provide information about the posterior funiculus, MEPs express the function of the anterior funiculus. SEPs have a higher rate of false positive signals and detect spinal ischemia with a certain amount of latency [215]. MEPs detect spinal ischemia within 2 min but depend on the anesthetic procedures [216]. An evaluation of MEPs and SEPs for 78 patients with thoracoabdominal aneurysms presented a sensitivity of 100% and a specificity of 94.2% [217].

An alternative to these techniques is near-infrared spectroscopy (NIRS). This technology is based on the oxygenation of the paraspinal musculature and the concept of the collateral network. This concept states that the spinal cord is perfused by the same collateral network as the paraspinal musculature [218]. The advantage of this technology is that application is much easier than in other techniques and prolonged measurement over a number of days is possible. The disadvantage is that NIRS has not been evaluated in larger studies. Existing results have been obtained from animal studies [219] and limited clinical data [218, 220]. However, two larger studies comparing NIRS technology with neurophysiological monitoring are currently underway [221, 222].

#### Preoperative coiling of spinal arteries

A relatively new concept is to coil spinal arteries before the main operation. This action mimics the staged concept of endovascular procedures. Based on the collateral network concept, occlusion of segmental spinal arteries should also increase other collaterals. A large randomized multicenter trial addressing the use of coiling in this context is currently underway [222].

### Therapy of the spinal ischemia

The choroid plexus produces 400–600 ml of spinal fluid per day with a time-dependent rate of 0.2–0.7 ml/min [223]. Occlusion of spinal arteries, hypotension, inflammation, etc. induces a hypoperfusion of the spinal cord. This results in an edema that aggravates the hypoperfusion, causing a penumbra. The spinal cord is surrounded by spinal fluid and the meninges. These meninges are not extensible, which results in a further hypoperfusion of the penumbra, creating a vicious circle. Aortic clamping induces elevated liquor production. Considering these aspects, the following recommendations are given:

- lowering the intraspinal pressure
- elevating the systemic perfusion pressure
- raising the maximal oxygen exhaustion

These recommendations can be followed by performing spinal drainage, elevating blood pressure, reducing central venous pressure and raising hemoglobin to 10 g/dl.

#### Spinal drainage

Spinal drainage can improve spinal ischemia [199, 224, 225]. Most guidelines recommend the immediate use of spinal drainage [6, 43, 207]. The target intraspinal pressure should be < 10 mm Hg [202]. The physiological background is that the perfusion pressure of the spinal cord is a direct function of the mean arterial pressure minus the intraspinal pressure (alternatively, the central venous pressure).

There is no consensus concerning the recommended amount of drained spinal fluid. In a comparative study [214] concerning volume-dependent (≤ 25 ml/h) and volume-independent drainage, there were no neurological differences found between the groups. In the volume-independent group, the rate of postoperative blood-tinged spinal fluid was higher.

#### Elevation of the systematic pressure and enhancing cardiac output

The mean arterial pressure should be approximately 80–100 mm Hg. Intraspinal pressure below 10 mm Hg indicates perfusion pressure of the spinal cord of approximately 70 mm Hg. Vasoconstrictors are advantageous in volume therapy for avoiding an elevation of the central venous pressure [202].

Importantly, in an acute aortic dissection, elevated systemic blood pressure can deteriorate the aortic dissection, which requires careful management.

#### Increase of the hemoglobin level ≥ 10 g/dl

**Fig. 10:**
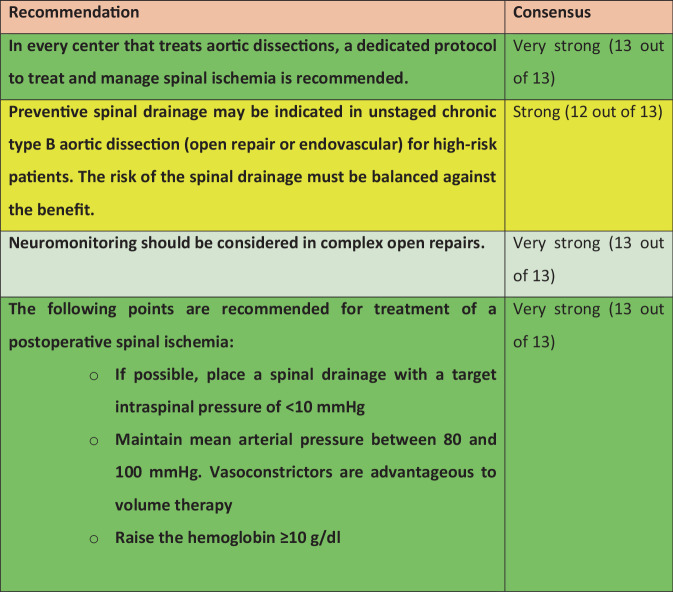
Recommendation 7: spinal ischemia

The purpose is to raise the maximal oxygen exhaustion. Multiple consensus conferences and expert recommendations advocate maintaining a hemoglobin level ≥ 10 g/dl for at least 72 h postoperatively or until the removal of the spinal drainage ([77, 202, 207, 226, 227]; Fig. 10).

## Rehabilitation

### Basic recommendations for rehabilitation

Rehabilitation includes measures aimed at eliminating or alleviating the physical, psychological and social consequences of a disability or restricted activity. In type B aortic dissections, rehabilitation should always be considered. This rehabilitation can be performed on an inpatient or, if medically justifiable, on an outpatient basis. Cardiological or angiological rehabilitation options are primarily considered for type B aortic dissections. Rehabilitation applications should contain the indication for rehabilitation (rehabilitation needs) and the objective of the treatment (rehabilitation objectives); additionally, applications should indicate the suitability for rehabilitation (rehabilitation ability). The ability to perform rehabilitation in cardiological/angiological rehabilitation clinics is essentially only limited if self-care is physically, psychologically or cognitively impossible (e.g. Barthel index < 60) or if geriatric patients have a high degree of frailty. In the latter case, subject-specific geriatric rehabilitation should be sought.

If the need for rehabilitation, the rehabilitation goals and the ability to rehabilitate are given, and if the referring specialist clinics believe that the patients are stable and resilient with respect to the aftercare findings, then cardiological, angiological or, if necessary, geriatric rehabilitation should be performed to enable patients to participate in everyday life (social, professional and societal participation). Subsequently, rehabilitation after type B aortic dissection of the aorta can be performed independently of the previous therapeutic procedure.

In rehabilitation, there are only a few specific approaches that relate solely to type B aortic dissection. However, excerpts from guidelines are available that can more broadly describe the postinterventional or postoperative possibilities and limits of rehabilitation in connection with aortic diseases. In particular, the S3 guidelines for cardiological rehabilitation (LL-KardReha) [228] in German-speaking Europe (Germany, Austria, Switzerland [D-A-CH]) directly addresses this topic and can be applied to the procedure for a type B aortic dissection.

Currently, studies on rehabilitation for type B dissections alone are very limited. In many studies, the term “rehabilitation” is not interpreted as a multimodal treatment concept but rather solely as a training therapy. The data in these studies mainly relate to rehabilitation after aortic syndromes with interventional or surgical treatment. Therefore, recommendations for the rehabilitation of patients after dissection of the aorta must be used regardless of the localization, treatment and the anatomically functional stage classifications (Stanford A, B; DeBakey I, II, III). Study results on rehabilitation and physical training after type A dissection or other aortic diseases must also be used for type B dissection. The study results show that rehabilitation and dosed physical training can be carried out safely [229–231] for patients with aortic syndromes and that a benefit can be achieved in terms of physical performance and quality of life. In the case of uncomplicated type B aortic dissections, careful training and mobilization measures immediately after the acute event can also be effective [94, 232].

However, the functional limitations resulting from the dissections can vary widely. Therefore, attention must be paid not only to purely organic limitations but also to the frequently observed psychosocial effects of the disease [233].

In the context of rehabilitation after type B dissections, the focus should, therefore, be on the patients’ health-related quality of life. The determination of psychological consequences of illnesses such as anxiety, adjustment disorders, depression and post-traumatic stress disorder (PTSD); and the provision of general patient training for avoiding risky behavior by the medical and psychological service of the rehabilitation facility should be part of every rehabilitation effort.

### Drug setting during rehabilitation

The most important medical aspect during rehabilitation for type B dissections is strict blood pressure control at rest and under stress. In subacute (15–90 days) and chronic (> 90 days) dissections, resting blood pressure should always be < 140/90 mm Hg; ideally, blood pressure should be < 130/80 mm Hg [43].

Treatment should primarily include beta-blockers as they reduce aneurysmal degeneration of the dissected aorta and the need for surgical procedures [43, 234]. Calcium antagonists have demonstrated improved survival rates in patients with type B dissections [88]. Both angiotensin‑1 receptor antagonists (losartan) and general blockades of the renin-angiotensin-aldosterone system (RAAS) have been shown to slow aortic dilation [235, 236] and the rate of events after surgery [237] in patients with Marfan syndrome. Accordingly, a beta-blocker should be selected as basic treatment in combination with a calcium antagonist; additionally, a further combination with an angiotensin‑1 receptor antagonist is beneficial for drug-based blood pressure control in the context of rehabilitation for type B dissections. Treatment-resistant arterial hypertension is not uncommon after an aortic dissection. Therefore, extended antihypertensive treatment regimens should be used [238].

### Training intensity during rehabilitation

Physical performance is often limited in patients with type B dissections, regardless of the therapy administered at the start of rehabilitation [229]. The feasibility of stress tests, e.g. a stress ECG or spiroergometry, was investigated in two smaller studies in patients with type B dissections [229, 239]. Complications arising directly from the examination were not reported in the studies. The data suggest that stress tests can safely be carried out to control training or in the follow-up care. Additionally, the reduced physical ability of patients with type B dissections was confirmed.

If such stress tests are performed to control training, the blood pressure behavior should also be taken into account in addition to recognizing stress-related complaints (e.g. chest complaints, dyspnea, arrhythmias, etc.). During aerobic exercise, there is usually only a moderate increase in systolic blood pressure from 140 to 160 mm Hg. Values of > 180 mm Hg are more likely to be reached at submaximal or maximal exertion. Exercise tests should be stopped when the patients’ systolic blood pressure reaches 160 mm Hg.

However, such stress tests should only be performed after a detailed risk stratification that uses imaging methods and after consultation with the referring center.

The aim of the stress test is to introduce these patients to moderate endurance exercise with strict blood pressure monitoring. The recommendations for the stress test include limiting intensity to 3–5 MET (metabolic equivalent) or 12–13/20 on the Borg scale, RPE (e.g. brisk walking, cycling at about 15 km/h). This intensity of exertion, which is perceived as “somewhat strenuous,” is considered safe [240–244] and also leads to a permanent reduction in blood pressure and heart rate.

The metabolic equivalent (MET) can be used to compare different activities in terms of energy consumption. The MET corresponds to the conversion of 3.5 ml oxygen per kg body weight per minute in men: in women, the MET is 3.15 ml oxygen/kg/min. For ease of use, the MET of energy consumption can be derived from previously defined tables [240, 245].

The Borg scale is used to estimate the subjective perception of stress. The RPE received perception of exertion (RPE) value assigned to each exercise enables patients to assess how stressful the training may be perceived individually [246].

Competitive contact sports and isometric exertion with forced breathing should be avoided because they cause increased wall stress due to sudden and steep rises in blood pressure.

For type B dissections that are treated surgically, attention should be paid to incisional hernias and exercises with increased thoracoabdominal tension; additionally, mechanical stress should be avoided.

### Sociomedical assessment and professional reintegration

Minimal data have been found on the topic of professional reintegration regarding type B dissections [229, 247]. In the context of rehabilitation, the aim should be to professionally reintegrate patients that have jobs. This becomes difficult when vigorous physical activities (> 6 MET) have to be performed on a daily basis [248]. Additionally, particular attention must be paid to static holding work, heavy lifting and strenuous movements with consecutive increases in blood pressure. Psychosocial stress or concomitant illnesses can make sociomedical assessment even more difficult. On the other hand, physical activities with light to occasionally moderate work components can often be reintroduced. Therefore, with each rehabilitation effort, measures for professional reintegration should be defined and, if necessary, implemented (e.g. internal solutions, benefits for participation in working life [249]) in collaboration with the social service of the rehabilitation facility.

### Older and geriatric patients with type B aortic dissection

The ability to cope with disease stressors decreases with age. However, this decrease not only applies to the respective disease itself; acute physical as well as psychological stressors can also lead to a destabilization of supposedly uninvolved organ systems. This decrease is a constant of the normal aging process both in “healthy” old people > 80 years of age and especially in people with a constellation of findings that is referred to as a “geriatric symptom complex” (immobility, tendency to fall, cognitive and affective deficits, malnutrition, vision and hearing loss, medication problems, etc.) [250]. The frailty syndrome presents pathologically reduced general muscle strength (sarcopenia) accompanied by weight loss and exhaustion.

The patient group of geriatric patients with type B aortic dissections poses a challenge towards determining indications and performing complex vascular procedures. One North American registry study showed that the degree of frailty correlates with the mortality of all common vascular surgical procedures [251] especially in open and endovascular aortic procedures [252]. Validated frailty indices can be used as prognostic parameters and are of great importance for therapeutic decisions and rehabilitation [253].

Typical geriatric-associated clinical problems already typically occur during acute treatment (e.g. delirium in up to 40% of patients with vascular interventions) [254]. It is therefore helpful and desirable to integrate locally available geriatric competence early in the peri-interventional/perioperative stage.

Essentially, a prolonged convalescence phase is to be expected due to acute medical complications as well as problems in regaining independence. As soon as acute vascular medical care after type B aortic dissections can be concluded, geriatric early rehabilitation provides patients with the necessary geriatric treatment team (physiotherapy, ergotherapy and, if necessary, speech therapy and psychological services). Institutions of geriatric early rehabilitation are specialist hospitals or specialist departments for geriatrics with a given rehabilitative geriatric structural quality. This measure leads to a reduction in mortality, an improvement in functional outcomes and a reduction in nursing home admissions [255].

### Summary

**Fig. 11:**
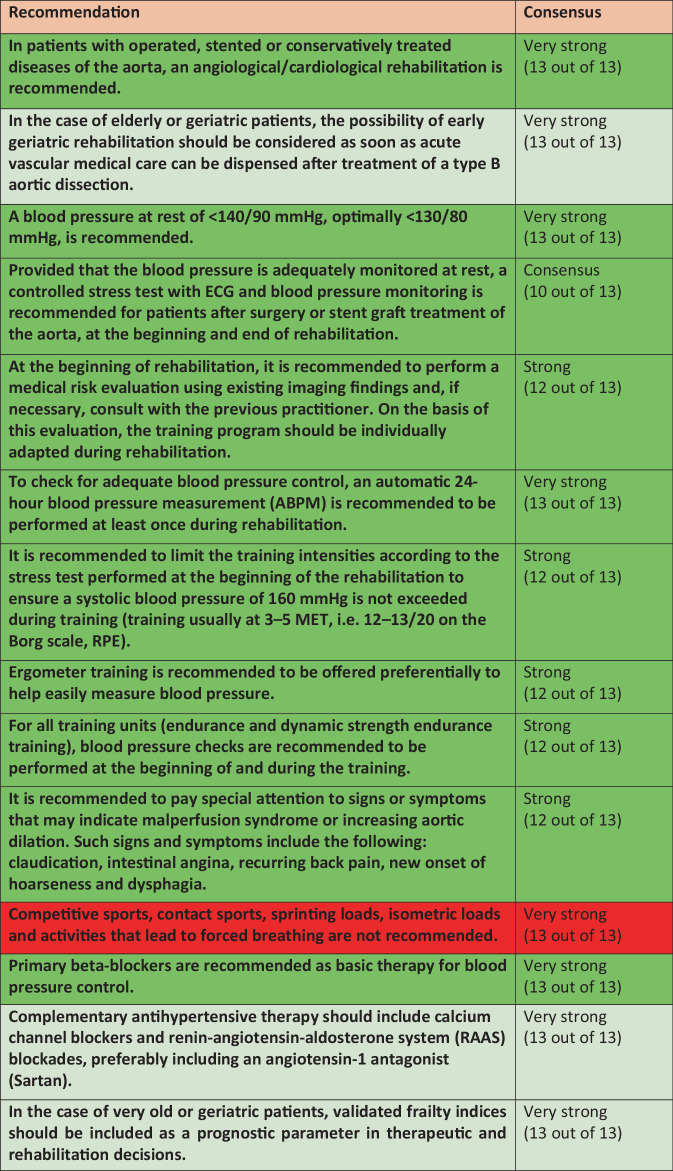
Recommendation 8: rehabilitation

The 2020 S3 guidelines on cardiological rehabilitation in German-speaking countries [228] summarize the recommended procedures for aortic syndromes and for all interventions in the aorta, including type B dissections. With respect to the recommendations made, consultations on these aspects helped to supplement and summarize previous guidelines; these additions are presented in the following Fig. 11.

## Psyche

### General comments and data regarding psychological reactions after aortic dissection

There are very few studies and publications that specifically address the association of type B aortic dissections with psychological reactions or illnesses. Therefore, consultation is required for studies on type A dissections on other cardiovascular diseases in connection with psychocardiology. These usually deal with the question of whether and how mental illnesses can be a risk factor for an acute cardiovascular event and how, conversely, cardiovascular events result in mental reactions or even illnesses. Previous research has made it apparent that an acute event such as a type B aortic dissection has psychological consequences that may limit quality of life more than physical illnesses or physical conditions [233, 243, 256]. Acute events such as type B aortic dissections may even lead to the occurrence of post-traumatic stress disorder (PTSD), for which patients require trauma-specific psychotherapy. There is an urgent need for research on the risk of PTSD after type A and type B aortic dissections [257–259]. Research has also identified that psychologically stressful situations and sleep disorders can act as triggering factors for aortic dissections [92, 260]. It is, therefore, important to include these psychosocial aspects in the treatment of aortic diseases and, if necessary, address these aspects therapeutically.

Adherence to taking the antihypertensive medication required for preventing recurrence in type B aortic dissections is also an important factor in connection with psychological comorbidity (e.g. depression) [261].

### Screening for psychological comorbidity after type B aortic dissection

The following conditions are the most common comorbidities of general cardiovascular events:

- depression
- anxiety disorder
- post-traumatic stress disorder (PTSD)

These potential comorbidities can be determined by asking patients specific questions in a medical consultation or by using screening instruments (Table 10).

**Table 10:** Questions and instruments for the diagnosis of mental disorders/comorbidities after a type B aortic dissection and/or other cardiovascular events (taken from [262])

Comorbidity	Screening questions for medical history	Standardized questionnaires
Depression	During the past month, have you often felt sad, depressed or hopeless? In the last month, have you had significantly less desire and pleasure in things that you usually enjoy doing?	Depression subscale of the Hospital Anxiety and Depression Scale (HADS) [263, 264] or the Patient Health Questionnaire (PHQ-9) [265, 266]
Generalized anxiety disorder	Do you feel nervous or tense? Do you often worry about things more than other people? Do you feel like you are constantly worried and not in control?	Hospital Anxiety and Depression Scale (HADS) [263, 264] anxiety subscale or PHQ Generalized Anxiety Disorder 7 (GAD-7) module [267, 268]
Post-traumatic stress disorder	Do you suffer from intrusive, stressful thoughts and memories of a serious event (images, nightmares, flashbacks)? (The event may also be a cardiac event or its treatment)	Impact of Event-Scale – revised (IES-R) [269]

If the screening through anamnesis or standardized questionnaires suggests a suspicion of psychological or psychosomatic comorbidities, therapeutic consequences or referrals to other specialist groups (psychosomatics, psychiatry, psychotherapy) for further diagnostics and, if necessary, (psycho)therapy are required.

### General recommendations for psychological comorbidities associated with a type B aortic dissection

**Fig. 12:**
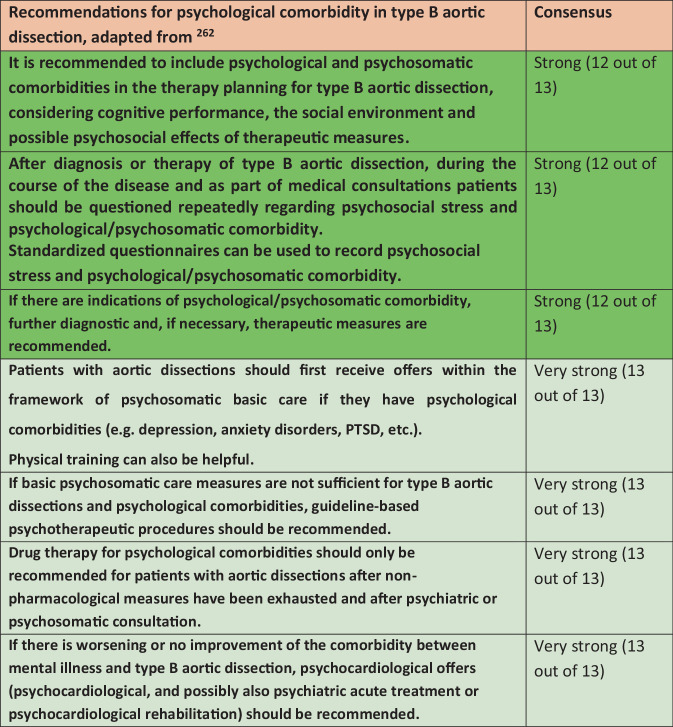
Recommendation 9: Psyche

The following recommendations for psychological comorbidities are presented from the perspective of general psychocardiology and represent expert opinions and good clinical practice. The recommendations are based on systematic research for the National Care Guideline (NVL) for Heart Failure and Chronic CHD, [262, 270] a position paper on the importance of psychosocial factors in cardiology [256] and the S‑3 guidelines on cardiological rehabilitation ([228]; Fig. 12).

## Unmet needs

Evidence is increasing in the area of type B aortic dissection. Nevertheless, there are no randomized studies to make level Ia recommendations. A problem is certainly the low incidence and the small number of cases in the individual centers and the great heterogeneity of the disease and the morphological characteristics. However, some points were considered necessary by the guideline group to generate evidence in the coming years. These are in the first line:

- What influence does early mobilization have on the aortic outcome and how great is the associated morbidity?
- Which risk group benefits from early endovascular treatment in acute, uncomplicated aortic dissection in terms of reducing late aortic events? How can this cohort be defined in more detail?
- What influence does IVUS have on the sizing and assessment of the true and false lumen in acute complicated aortic dissection?

## Declarations

For this article no studies with human participants or animals were performed by any of the authors. All studies mentioned were in accordance with the ethical standards indicated in each case.

The supplement containing this article is not sponsored by industry.
